# Enablers and barriers to using patient decision aids in early stage breast cancer consultations: a qualitative study of surgeons’ views

**DOI:** 10.1186/s13012-014-0174-0

**Published:** 2014-11-29

**Authors:** Mary Ann O’Brien, Cathy Charles, Peter Lovrics, Frances C Wright, Tim Whelan, Marko Simunovic, Erin Kennedy, Eva Grunfeld

**Affiliations:** Department of Family and Community Medicine, University of Toronto, 500 University Avenue, Fifth Floor, Toronto, ON M5G 1 V7 Canada; Knowledge Translation Research Network, Health Services Research Program, Ontario Institute for Cancer Research, Toronto, ON Canada; Department of Clinical Epidemiology and Biostatistics, McMaster University, Hamilton, ON Canada; St. Joseph’s Healthcare, Hamilton, ON Canada; Odette Cancer Centre, Toronto, ON Canada; Juravinski Cancer Centre, Hamilton, ON Canada; Mount Sinai Hospital, University Health Network, Toronto, ON Canada

**Keywords:** Patient decision aids, Decision support tools, Breast cancer surgery

## Abstract

**Background:**

For early stage breast cancer, randomized controlled trials (RCTs) have shown that patient decision aids (PtDAs), when used by surgeons, result in increased patient knowledge about options and different patient treatment choices as compared to standard care. Yet, recent data suggests that PtDAs are used by less than 25% of Canadian cancer physicians. We conducted a study to explore breast cancer surgeons’ views on enablers and barriers to the use of PtDAs in their practice.

**Methods:**

Purposeful sampling was used to select breast cancer surgeons in three Ontario health regions to participate in semi-structured interviews. Inductive coding and the constant comparative method were used to identify the main themes.

**Results:**

Twenty-two surgeons (79%) agreed to participate (median age, 50 years; 9 (40%) female). Surgeons practiced in academic (*n* = 7, 32%) or community (*n* = 15, 68%) hospitals. Fourteen surgeons were aware of PtDAs, nine had used a PtDA with patients as part of an RCT, and six had developed their own informal PtDA for use in their practice. Enablers of informal PtDA use included surgeon exposure during training and surgeon perceived need for a systematic approach when communicating risks and benefits of surgical treatments with patients. Barriers to formal PtDA use included high surgeon confidence in their verbal communication skills, surgeon belief that patients understood conveyed information, and difficulties embedding such tools in practice routines.

**Conclusions:**

Surgeons in this study valued systematic communication with patients. Several surgeons changed their practice to include formal or informal PtDAs provided they perceived there was a clear benefit to themselves or to patients. However, high surgeon confidence in their personal communications skills coupled with beliefs that patients understand conveyed information may be key barriers to PtDA uptake once surgeons have established communication routines.

## Background

Despite the importance of patient involvement in treatment decision making (TDM), there is evidence that cancer patients do not participate in TDM to the extent that they prefer. Several researchers have demonstrated marked incongruity between cancer patients’ preferences for involvement and their actual experiences in TDM [1-3]. Patient decision aids (PtDAs) have been developed to help patients participate in making healthcare decisions. They are described as “interventions designed to help people make specific and deliberative choices among options by providing information about the options and outcomes that is relevant to a person’s health status” [4]. PtDAs take various formats including paper-based, DVD, and web-based [4]. There has been a growing interest in PtDAs over the past 15 years as judged by the increasing number included in updates of a large Cochrane review [4].

Evidence from several systematic reviews demonstrates that PtDAs in a cancer setting can improve patient knowledge, patient satisfaction with the decision making process, or the decision itself and may have an impact on patients’ treatment choice [5-10]. In particular, PtDAs for women considering options for breast cancer surgery have been shown to have positive effects [5,6]. For example, in a randomized controlled trial (RCT), Whelan et al. demonstrated that women who received a PtDA had improved knowledge about their surgical options and more commonly chose breast-conserving treatment (i.e., a lumpectomy or partial breast removal plus radiation) over a mastectomy (i.e., complete breast removal) (94% vs. 76%, *p* = 0.03) [10].

Despite evidence of their effectiveness, uptake of PtDAs into clinical practice continues to be limited [11-13]. In a population-based study in Ontario, Canada, only 24% of general surgeons and oncologists used PtDAs in their practice [11]. The precise reasons for the lack of uptake of PtDAs into practice are unknown but are likely multi-factorial [12,14]. Harrison and colleagues studied the views of colorectal surgeons toward PtDAs and found that time restraints, characteristics pertaining to the patient, content of the PtDA, and concerns about impacting the patient-surgeon relationship were perceived barriers to the implementation of PtDAs in usual practice [15]. To better understand issues pertaining to uptake, we conducted a qualitative study in which we asked surgeons about their views and experiences with PtDAs in their surgical consultations with women with breast cancer. Specifically, we asked them to describe their treatment discussions with breast cancer patients, any experiences using PtDAs, and their perceptions of enablers and barriers to the use of PtDAs in surgical oncology consultations. The objective of this study was to determine the perceptions of surgeons of facilitators and barriers to the uptake of PtDAs in breast cancer surgical consultations.

## Methods

A descriptive qualitative design was used [16] as there is relatively little published evidence of factors affecting the uptake of PtDAs in surgical oncology practice. Participants were surgeons in full-time practice performing breast cancer surgery in three health regions in Southwestern Ontario. A maximum variation purposeful sampling strategy was used to create a diverse mix of surgeons in academic and community-based practices including surgeons with and without experience of using PtDAs, both male and female surgeons and those with varying years in practice. To create the sampling frame, a list of surgeons’ names and postal addresses was created from the publically available College of Physicians and Surgeons of Ontario (CPSO) website. In our sampling strategy, one of the health regions included surgeons who had previously participated in one of two RCTs of PtDAs [10,17]. Using the list of CPSO surgeons in each health region, key informants (two surgeon-leaders PL and FW) suggested potential participants. Surgeons were sent a mailed invitation signed by the surgeon-leader. Each surgeon’s office received one or two follow-up phone call(s) by the principal investigator (MAOB) to request an interview.

### Data collection

Surgeon demographic (age, gender, number of years after surgical residency training) and practice characteristics (type of hospital [community or academic], total number of surgeons performing similar surgery at the hospital, percentage of breast cancer patients in practice) were collected. Semi-structured interviews (telephone or in person) lasting approximately 40 min were conducted by an experienced interviewer (MAOB). A draft interview guide was created and included questions about potential enablers and barriers to adopting innovations in practice (i.e., characteristics of the innovation, patient, surgeon, practice, and hospital) as previously published by Grol and Wensing [18]. The interview guide was pilot tested for question clarity with two volunteer surgeons and modified as new lines of inquiry were identified during the interviews. All interviews were audio recorded and transcribed verbatim. The software program NVivo 9 (QSR International Pty Ltd) was used to facilitate coding and retrieval processes of data analysis.

### Data coding and analysis

A preliminary coding guide was developed. The concepts of “enablers” and “barriers” as described by Grol and Wensing [18] were used as sensitizing concepts to identify specific examples during the coding process [19]. We used the term *informal* PtDA to connote participants’ descriptions of surgeon-developed decision tools including hand-drawn diagrams designed to explain surgical options. We used the term *formal* PtDA to refer to aids evaluated in two previous RCTs [10,17]. We used an editing style of coding whereby each analyst read every line of each transcript and assigned codes to concepts [20]. The constant comparative method was used to inductively identify the main themes [21-23] from the coded transcripts. All study transcripts were coded by two analysts (MAOB, MHM) who compared their codes, developed themes, and reached agreement on the identified themes. All themes were reviewed by a third member (CC) of the study team.

#### Study rigor

Study rigor was addressed in three ways: 1) team involvement in the development of the coding scheme using an iterative process of developing, applying, and modifying the scheme; 2) development and use of decision rules to guide future coding based on agreement of how to resolve prior inter–rater discrepancies in coding; and 3) use of an audit trail [24] which included interview summaries and memos documenting all major decisions taken during the study.

The study protocol was approved by the Research Ethics Boards of the University of Toronto and Hamilton Health Sciences/McMaster University.

## Results

### Demographics and practice characteristics

Twenty-two surgeons (79% of those approached) agreed to participate (median age, 50 years; 9 (40%) female). The median number of years post-surgical training was 15 (range 2–28 years). Surgeons practiced in academic (*n* = 7, 32%) or community (*n* = 15, 68%) hospitals. The percentage of breast cancer patients in surgeons’ practices was estimated to be 20% (median, range 5%–100%).

### Awareness and use of PtDAs

Fourteen of 22 surgeons were aware of PtDAs (Table 1). Of these 14 surgeons, 13 had previously used a PtDA either as part of their consultation (11 surgeons) or had recommended one for patient use at home after the consultation (2 surgeons). Of the 11 surgeons who had used a PtDA during the patient consultation, nine had used one while participating in one of two RCTs [10,17]. Two of these surgeons continued to use this PtDA occasionally (approximately twice per year) for patients who had difficulty understanding their treatment options. Four of these surgeons created their own hand-drawn modified version of the PtDA used in the trial and used it consistently for each patient. One additional surgeon said that their verbal description of surgical options was modelled upon the PtDA used during a previous RCT. Two surgeons, while unaware of published PtDAs, had created their own informal PTDA after seeing one used by a mentor during their surgical training.

**Table 1:** **Surgeon awareness and use of patient decision aids (PtDAs) for breast cancer surgery**

**Awareness and use of PtDA**	**Number of surgeons (%)**
Aware of PtDAs	14 (64)
Previously used formal PtDA	9 (41)
Currently uses formal PtDA	2 (9)
Uses informal PtDA	6 (27)

*Abbreviations: PtDA* patient decision aid.

### Surgeons’ views of decision making processes

During the interviews, the surgeons described patient and surgeon involvement in decision making related to the choice of surgical procedure. In general, most surgeons described a collaborative process to decision making in which the surgeon provided information about options and both parties expressed their preferences. For example,

“I think it’s important to give the woman the information and for them to make the decision … I do give them some of my experience”.

Surgeon 20, participated in RCT (decision board—usual care group)

“I think you want someone to help you sort through the issues and I think that’s the importance of, well not just ‘these are your two choices, take a couple of minutes I will be back, tell me which one you want’”.

Surgeon 8, unaware of PtDAs

When the surgeons perceived that both options were equally viable, the surgeons expressed that women should make the surgical choice.

“I think the patient has the right to make the choice and they should make the choice because I tell them whatever they choose I will be happy to do … because you are the one that has to live with your decision”.

Surgeon 18, used standard board PtDA during RCT, uses informal PtDA

While most surgeons viewed decision making as a collaborative process, only some surgeons used a PtDA. In the following sections, we describe surgeons’ views of enablers and barriers to the use of formal or informal PtDAs in practice.

### Themes related to surgeon use of formal or informal PtDAs

Five themes emerged that were related to surgeon use of PtDAs. These were 1) surgical training and mentorship shapes how surgeons communicate with patients; 2) communication routines help ensure that important issues are remembered and stated; 3) there may be less “buy-in” to change communication routines as compared to surgical techniques; 4) surgeons’ views of the nature of PtDA outcomes inhibit their use; and 5) high confidence in surgeon’s own communication skills curtails searching for and using PtDAs.

#### Surgical training and mentorship shapes how surgeons communicate with patients

Surgeons described the influence of their surgical training, both in being exposed to informal PtDAs used by their mentors and the extent that they had opportunities to practice communication skills with patients during clinics in addition to the training received in the operating room. Several surgeons who were in practice for less than 5 years described that seeing a mentor use an informal PtDA influenced their use of a PtDA when they began independent practice.

“You pick and choose what aspects of practice you would like to emulate, and what you would like to incorporate into your own practice. So I worked with a couple of surgeons who did that routinely, the drawing part, and it made sense to me in terms of how you explain it, you have that consistency. So that’s why I choose to do it in that fashion”.

Surgeon 1, created own informal PtDA

In addition to describing their use of PtDAs during training, the surgeons related that surgical residency training has changed over time with a greater emphasis on practicing communication skills with patients in an office setting. Several surgeons who had been in practice for longer than 20 years relayed that, when they were residents, most of their time was spent in the operating room with little time spent in practicing communication skills with patients in office-based clinics. These surgeons discussed having to learn how to communicate to patients largely on their own when they assumed independent practice. The surgeons said that, currently, surgical residents spend time in clinics talking with patients although there may be variable encouragement by mentors to do so.

#### Communication routines help ensure that important issues are remembered and stated

All surgeons discussed the importance of developing communication routines with patients. They described that, early in their careers as independent surgeons, they did not feel comfortable with the way they explained information to patients. They spoke of taking a long time during consultations or being less than clear when explaining options to patients. Several surgeons who were relatively new in practice (less than 5 years) described that an informal PtDA that they had adopted after seeing one used by a mentor during residency helped them develop an effective communication routine. Other surgeons who had adapted a PtDA after using one during an RCT agreed. Having a pattern or routine way of explaining information helped to ensure that he/she would not forget to give patients important information about the risk of recurrent cancer or outcomes of treatment. For example,

“I think a lot of the things in medicine, be it surgery or whatever, is getting into a pattern of doing things the same way every single time so that you won’t forget something. And when I go through it I do my picture, I do the exact same thing every time, I know I’m not going to miss one of the risks or one of the outcomes or one of the side effects”.

Surgeon 13, used computer and standard board PtDAs during RCT, uses informal PtDA

Surgeons who were unaware of PtDAs corroborated the importance of communication routines. They commented that they developed their pattern of talking with patients through experience during practice. They said that a PtDA might be useful for someone new in practice. One surgeon who had been in practice for several years stated,

“I think it’s a good aid when you are first coming out in practice to make sure you have covered all your bases … But … what makes an expert an expert is pattern recognition. Most people that have been out long enough to have dealt with this problem, sort of know what to say and what not to say. And it may not be as big of a need with them as somebody who is freshly out [in practice] who are still trying to develop the way they are going to tell people their problems”.

Surgeon 6, never used a formal or informal PtDA

While the development of communication routines was important to surgeons, PtDAs did not necessarily fit well with established routines. The surgeons who had used a PtDA during an RCT said that it was difficult to fit the aid into their communication preferences and routines. They experienced difficulties adapting the PtDA to suit their own communication style. A surgeon who had participated in an RCT said,

“Even though I spend time with patients, it [using a PtDA] still is difficult because now you have a scripted message and doing scripting is difficult. Because everyone is going to do things differently. You can see the same play at [a] festival and you can see it on the big screen, it’s the same play but it’s not the same”.

Surgeon 15, used standard board PtDA during RCT

Another surgeon agreed saying,

“For me I find it [PtDA] very cumbersome and it depends on your personality too. I’m not the one who picks up a paper and explain things like that. So usually how I do [it] is I have my own, I call [it] my own decision board when I see a patient, this is what I go through and then I usually draw a couple of the drawings”.

Surgeon 18, used standard board PtDA during RCT, uses informal PtDA

#### Less “buy-in” to change communication routines as compared to surgical techniques

Several surgeons commented on instances when they had made changes to incorporate a new surgical technique in their everyday practice. They appeared to be less motivated to make changes to their communication routines.

“I think if it’s a surgical technique, if it’s technical driven, we’re all on it, it’s like yeah, let’s try it, let’s do it. Or show me about that or how does that work? And then we’re interested. When it’s like well now you can use this to discuss this option with your patient, I don’t think people are going to dismiss it, I’m just not certain what the buy- in is going to be”.

Surgeon 13, used computer and standard board PtDAs during RCT, uses informal PtDA

Nevertheless, several surgeons had made changes to their communication routines by adapting a previously developed formal PtDA. However, the above quote and the one that follows suggest that the nature of change, i.e., surgical techniques versus communication skills, may be an issue that may influence the adoption of an innovation.

“[Surgeons] will be more motivated to seek out improvements in surgical techniques than they would be with communication skills”.

Surgeon 19, unaware of PtDAs

#### Views of the nature of PtDA outcomes inhibit their use

Several surgeons indicated that the outcomes of PtDAs were not compelling enough to change their practice. They seemed to be more interested in innovations in surgical techniques that might affect surgical outcomes. For example, a surgeon who was previously unaware of PtDAs said,

“Something like this [PtDA] it might make my job a little bit easier or it make it a little easier to get that information translated across. But I don’t see it as making a big difference in the outcome or the practices. So I’ve been less interested or less enthused about going and looking for the information”.

Surgeon 7, unaware of PtDAs

“You can show all the positive results but it’s hard to get people to change their practice and their way of doing things, for that kind of thing where it doesn’t matter. But … if all of a sudden, a study came out that appendectomy was dangerous, well then of course everybody is going to change their practice but this isn’t quite as dramatic”.

Surgeon 14, uses informal PtDA

#### High confidence in one’s own communication skills curtails searching for or using PtDAs

Several surgeons commented that they were confident in their ability to communicate information to patients. They did not perceive a need to look for or use a PtDA.

“I think that I do an okay job right now so I haven’t really sought out to find this [PtDA], which would be different than when we were starting doing sentinel nodes [surgical technique]. I made a point of learning [about sentinel lymph node biopsy] myself out in the community … because I thought that was a significant change in practice”.

Surgeon 7, unaware of PtDAs

Related to the surgeon’s confidence in his or her communication skills was the perception that patients understood information about treatment options conveyed by the surgeon. For example,

“I am kind of focussed and cover everything and then at the end of our discussion most of the time the patients say, well they checked through [and said], ‘I have no questions left’”.

Surgeon 9, participated in RCT (standard board—usual care group)

Many surgeons shared this view. For example,

“Typically I feel my patients are very well informed and they know what is going on and they know what their choices are. I have never felt the urge to look for anything [PtDA]”.

Surgeon 8, unaware of PtDAs

“Well I think the patient is informed. We did not find patients don’t understand or patients have any unanswered questions with what we are doing. Obviously always there can be improvement to any practice, but what we are currently doing, I think patients are quite happy with that”.

Surgeon 12, unaware of PtDAs

### Innovation-related enablers and barriers

In addition to the surgeon-related enablers and barriers discussed in the previous section, the surgeons also indicated that the characteristics of the innovation itself also influenced their use of formal or informal PtDAs. The innovation-related enablers were derived from surgeons’ experiences with the informal PtDAs; their views on barriers came from using a formal PtDA in one of two trials [10,17].

#### Innovation-related enablers

A key enabler was the ease of tailoring the informal PtDA to the individual patient. Surgeons described writing directly on the tool during the consultation. Other positive features included having all pertinent information about treatment options on one page. Surgeons said it was easy to photocopy the tool (including their handwritten comments) and give one copy to the patient and place the other copy in the office patient record.

#### Innovation-related barriers

The surgeons also described two key barriers related to the continued use of the formal PtDA first used during an RCT. The first barrier pertained to physical aspects of the PtDA, while the second related to the information contained in the aid. Surgeons who participated in a previous trial thought that either the computer version or a large “decision board” was too awkward to use both during and after the trial. The surgeons reported difficulty with obtaining information on the computer version while others said the decision board was too large to use in a small consultation room. Several surgeons commented that surgical practice was changing and that the information on the PtDA was out of date as it did not include current information such as the newer technique of sentinel lymph node biopsy.

## Discussion

Almost two-thirds of the surgeons in the present study were aware of PtDAs and just under half of the surgeons had used a PtDA during their consultations with breast cancer patients with just over one-quarter using an informal PtDA regularly. An interesting finding of the present study is that several surgeons adapted the formal PtDA used while participating in a trial and continued to use it in their everyday practice with patients nearly 10 years later. It is likely that for several surgeons, participation in the clinical trial encouraged the early adoption of the PtDA, albeit with subsequent modifications to better fit with their style of practice. By participating in the trial, the surgeons had the opportunity to practice using the PtDA. This process may be similar to the concept of “trialability” of an innovation [25,26]. Grilli and Lomas suggest that trialability can affect the uptake of clinical practice guidelines [26]. However, trialability may not always be important in adoption of an innovation. Simunovic et al. reported that trialability was not associated with increased adoption of an innovation in colorectal cancer surgery [27].

There were several similarities between the adapted informal PtDA and the informal PtDAs developed by two surgeons who had not participated in a trial. All tools were on one page and could easily be photocopied for the patient and placed in her chart. Another aspect was ease of tailoring the aid to the individual patient. The surgeons liked being able to write on the tool as issues came up during discussion with the patient. Finally, our study has identified the influence of mentors during surgical training as a factor in the surgeons’ use of informal PtDAs in independent practice.

Several of the findings from the present study are similar to those in the literature [12,28-31]. For example, Légaré et al. in a systematic review of enablers and barriers to shared decision making found that most frequently occurring facilitators were professional motivation and beliefs that patient outcomes would be improved [31]. In the present study, the surgeons mentioned the relevance of patient outcomes but that those related to communication outcomes were not as compelling as other outcomes such as those pertaining to surgical techniques. Several surgeons indicated that they were more motivated to change practice behavior that focused on surgical techniques rather than on communication skills as the latter was seen as more peripheral to their role as surgeons, although this view was not held by all surgeons. A striking difference between the findings of the Légaré review and our study was that only one surgeon indicated that lack of time was a barrier to use of a PtDA.

Elwyn et al. interviewed 57 health professionals about referring patients with health problems including breast cancer and prostate cancer to online patient decision support tools [30]. They found that these professionals were largely unwilling to refer patients to use these tools and did not change their practice. In their study, health professionals were asked to refer patients to web-based decision support tools whereas in our study surgeons were asked about their own use of such tools during their consultations with patients. Nonetheless, in both studies, the importance of practice routines is a common finding. Caldon et al. 2011 [12] also reported several concerns of health professionals with respect to PtDAs. These included threats to personal autonomy, concerns that patients would ask too many questions, and disagreement with the content of the tool [12]. None of the surgeons in our study expressed concerns about threats to their autonomy although several surgeons emphasized that it was their role to communicate surgical options effectively to patients and to ensure their understanding of these options and their risks and benefits.

In the present study, we used the assessment of barriers and enablers to practice change described by Grol and Wensing [18]. Yet, Elwyn et al. have criticized the use of the “many barriers” approach to the adoption of decision support tools into practice [14]. Based on a conceptual analysis of previous research of decision support tools using the Normalisation Process Model, Elwyn et al. [14] contend that current research has largely focused on processes whereby decision support tools need to fit within the normal conduct and purpose of the clinical encounter, the roles of the participants, and the legitimate roles of shared decision making [14]. They suggest that researchers should also consider the impact of decision support tools on organizational resources. Others also suggest that organizational resources and partnerships are important for the uptake of complex interventions in practice [32,33]. While organizational resources are likely to be important for innovations of greater complexity, our findings suggest that the adoption of a PtDA was largely dependent on the views of the individual surgeon.

One of the findings of the present study was that the surgeons who had either adapted or developed a PtDA had created a simple one-page tool on which the surgeon could write specific information that was relevant to the particular patient. This is similar to the research of Elwyn et al. on the use of “decision grids” [34], one-page tools that are evidence-based and can be used within the patient-professional consultation.

### Strengths and limitations

#### Strengths

This study examined the views of a diverse group of surgeons who provide care for patients with breast cancer. Surgeons practiced in both academic and community settings in both urban and rural areas. We included both surgeons who were relatively new to practice (within 5 years) as well as more established surgeons. Approximately half of the surgeons had been exposed to a PtDA as part of an RCT, and thus, we were able to examine what happened to the real-life use of the PtDA in the intervening years.

#### Limitations

We were not able to sample all surgeons in the original RCT as we did not have access to their identities due to privacy concerns. As such, the extent to which their views would be similar to those of surgeons in the present study is unknown. Nevertheless, we did purposefully sample surgeons from the same study region as the original trial. Nearly 10 years had elapsed since surgeons participated in the original trial. Several surgeons commented that they had difficulty remembering all features of the PtDA but freely discussed features they did remember. However, their views of their current practice and use of informal PtDAs were unlikely to be affected by recall problems. Last, surgeon-leaders in each community contacted surgeons to tell them about the study. It is possible that these surgeons’ opinions are different from surgeons who were not contacted.

## Conclusions

While surgeons valued systematic methods of communication with patients, formal PtDAs did not fit well with their usual information-giving patterns and led several surgeons to adapt PtDAs. We suggest that surgeons can integrate informal PtDAs into their practice when such aids are perceived to add value to their role as surgeons, facilitate tailored communication with patients, and are easily integrated into practice routines. Yet, high surgeon confidence in their communications skills coupled with beliefs that patients understand conveyed information are key barriers to PtDA uptake once surgeons have developed communication routines in their daily practice settings.

## Abbreviations

TDM: Treatment decision making
PtDA: Patient decision aid
CPSO: College of Physicians and Surgeons of Ontario
